# Arterial Stiffness and Pulse Wave Reflection Are Increased in Patients Suffering from Severe Periodontitis

**DOI:** 10.1371/journal.pone.0103449

**Published:** 2014-08-01

**Authors:** Yvonne Jockel-Schneider, Inga Harks, Imme Haubitz, Stefan Fickl, Martin Eigenthaler, Ulrich Schlagenhauf, Johannes Baulmann

**Affiliations:** 1 Department of Periodontology, University Hospital Wuerzburg, Wuerzburg, Germany; 2 Department of Periodontology, University Hospital Muenster, Muenster, Germany; 3 Clinic of Internal Medicine II, University Hospital Schleswig-Holstein, Luebeck, Germany; University of Perugia, Italy

## Abstract

**Aim:**

This single blind cross-sectional study compared the vascular health of subjects suffering from severe chronic periodontitis, severe aggressive periodontitis and periodontal healthy controls by evaluating pulse wave velocity (PWV), augmentation index (AIx) and pulse pressure amplification (PPA).

**Material and Methods:**

In a total of 158 subjects, 92 suffering from severe periodontitis and 66 matched periodontal healthy controls, PWV, AIx, central and peripheral blood pressure were recorded using an oscillometric device (Arteriograph).

**Results:**

Subjects suffering from severe chronic or aggressive periodontitis exhibited significantly higher PWV (p = 0.00004), higher AIx (p = 0.0049) and lower PPA (p = 0.028) than matched periodontal healthy controls.

**Conclusions:**

The results of this study confirm the association between periodontal inflammation and increased cardiovascular risk shown by impaired vascular health in case of severe periodontitis. As impaired vascular health is a common finding in patients suffering from severe periodontal disease a concomitant routine cardiovascular evaluation may be advised.

## Introduction

The association between oral inflammation and the risk for myocardial infarction or stroke has been firstly described already more than two decades ago [1], [2]. Ever since, a steadily increasing number of studies and reviews have firmly established a positive association between atherosclerosis and periodontal inflammation [3]–[5]. Preliminary intervention studies were able to prove the positive influence of various periodontal therapeutic measures on endothelial dysfunction, e.g. sub- and supragingival debridement [6], [7], debridement supplemented by systemic antibiotics [8] or debridement in conjunction with local antibiotics [9]. For the evaluation of endothelial dysfunction, the first measurable stage of developing atherosclerosis, in these studies usually the flow-mediated dilatation FMD (endothelium-dependent) or the nitroglycerin-mediated dilatation (endothelium-independent) of the brachial artery, have been recorded. While the validity of data obtained by FMD and nitro-glycerine-mediated dilatation has been verified by various trials, both methods require a very high level of examiner training to avoid faulty measurements and are time-consuming and expensive. Therefore the need for less expensive and clinically less demanding alternatives for routine vascular recording lead to the re-evaluation of the well-established principle of pulse wave velocity (PWV), augmentation index (AIx) and central pressures as clinically highly relevant indicators of vascular health.

The measurement of arterial stiffness by pulse wave reflection may be regarded as a prognostic significant extension of conventional vascular diagnosis. PWV is a direct marker of arterial stiffness [10], [11]. Increased PWV is a strong predictor for future cardiovascular events and mortality in patients with and without diverse risk factors, as for example end-stage renal disease [12], patients with type 2 diabetes [13] hypertension [14], elderly people [15] or the general population [16]–[18].

**Figure 1:**
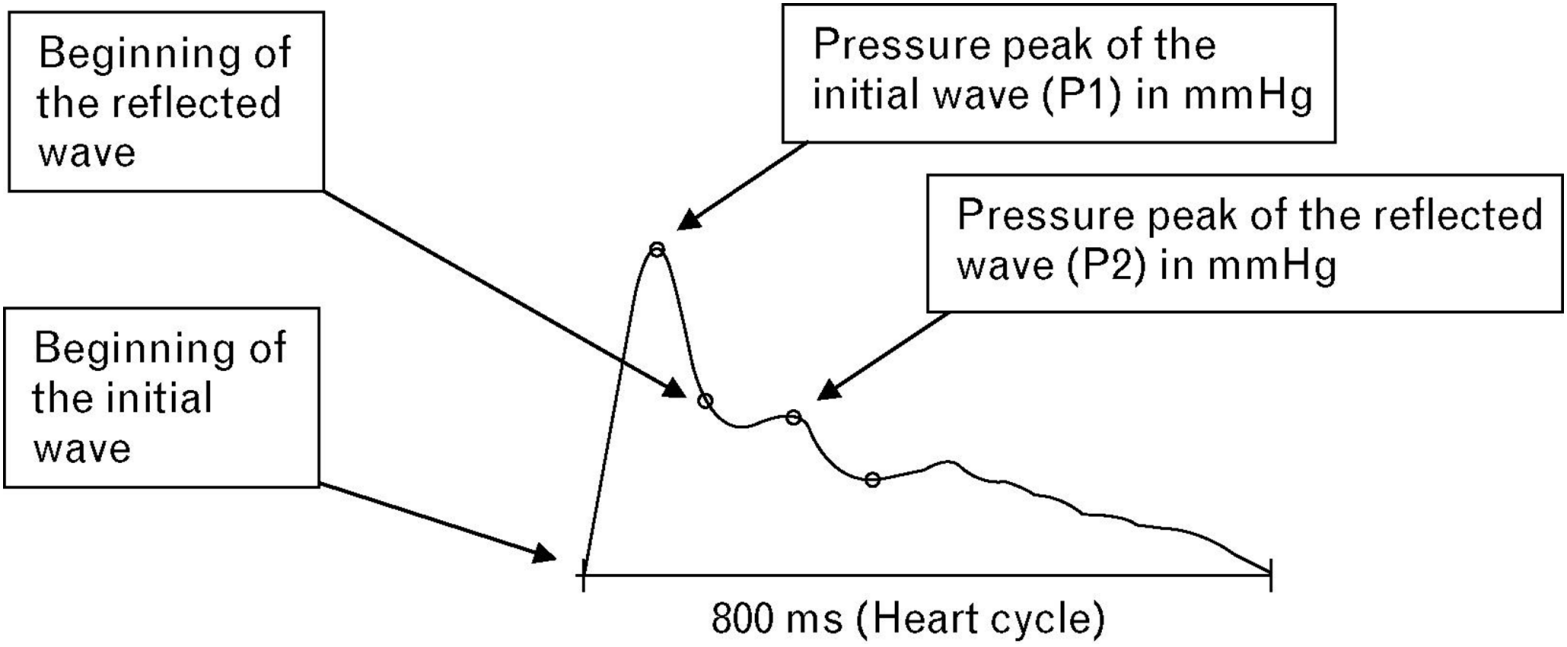
Original recording of an oscillometrically generated pulse wave (modified by Baulmann et al. 2008).

An indirect measure of arterial stiffness and a direct measure of pulse wave reflection is the augmentation pressure and the augmentation index [19]. Augmentation can be described as the effect of wave reflection on the aortic systolic pressure peak. Accordingly, augmentation is a measure for the additional pressure caused by pulse wave reflection stressing the left ventricle [20]. AIx may be calculated by dividing the augmentation pressure by the pulse pressure. In principle, the AIx may be obtained by calculating the quotient of the pressure peak of the initial and the reflected wave (Fig. 1 [19]. All parameters influencing the AIx subsequently also have an impact on the central blood pressure. The measurement of the pulse wave reflection therefore allows an estimation of the central blood pressure, and may differ substantially from the recordable peripheral blood pressure [19]. It correlates well with left ventricular mass in hypertensive and in normotensive young men [21] and is an independent marker for premature coronary artery disease [22]. In different populations e.g. patients with end-stage renal failure, the AIx is also an independent predictor of mortality [23].

Both, PWV and AIx, provide essential information on the status of the arterial vascular system. The prognostic significance of arterial stiffness is very high. Measurements of PWV and AIx calculations allow stratifying patients with a high risk profile for cardiac and cerebral events who might benefit from more intensive cardiovascular treatment schemes [19].

Recently a new recording system for the assessment of vascular function on the basis of recorded pulse wave data has been developed, the so called Arteriograph. The Arteriograph was the very first validated device that allowed the measurement of PWV, AIx and central pressures by analysis of the oscillometric pressure curves registered on the upper arm with a single pressure cuff [24]. The principle of the oscillometric method is based on plethysmography and registers oscillometric pulsatile pressure changes in the brachial artery. Pressure fluctuations in the brachial artery are revealed by a pressure sensor in the cuff passing the recorded data to a computer for further analysis and computation of pulse pressure waves (Fig. 1). PWV is calculated in m/s for a given patient by relating the recorded time difference (ms) between the onset of the first pulse wave and the onset of the reflected pulse wave to the measured distance between the jugulum and the symphysis. The AIx corresponds to the pressure difference (amplitude difference; P1–P2, Fig. 1) between the first and second wave in relation to the pulse pressure (PP). The Arteriograph calculates the AIx based on a fixed formula and thus provides the aortic AIx without applying a transfer function [24].

### Aim

The aim of this cross sectional study was to assess the cardiovascular status of patients suffering from severe aggressive or chronic periodontitis by recording pulse wave velocity, aug­men­tation index and central blood pressure with the Arteriograph and compare it to data recorded from matched periodontal healthy controls.

## Materials and Methods

### Study design

This investigation was designed as a single blind cross-sectional trial. The study protocol, prepared in accordance with the declaration of Helsinki of 1973 and meeting the GCP criteria, was approved by the ethics committee of the University of Wuerzburg. All subjects included have signed the informed consent. A positive vote of the ethics committee of the University of Wuerzburg from 2009 (file #39-09) is easily available (e.g. on request via mail to the corresponding author).

### Study setting

Study subjects were screened and recruited from individuals seeking dental care at the School of Dental Medicine of the University of Wuerzburg.

### Inclusion criteria

Patients suffering from untreated severe generalised chronic or aggressive periodontitis were eligible for participation in test group. The diagnosis of generalised severe chronic or aggressive periodontitis was based on the classification criteria of the International Workshop for a Classification of Periodontal Diseases and Conditions [25].

The severity of disease expression was categorized according to the proposal of the working group of the Centers for Disease Control (CDC) and the American Academy of Periodontology (AAP) [26].

In order to restrict inclusion only to subjects with extensive periodontal lesions study patients additionally had to meet the following criteria: Patients with clinical detectable attachment loss ≥6 mm in a minimum of two different sextants and a minimum of six interproximal sites on six different teeth were eligible for inclusion in the test group.

Individuals exhibiting minor periodontal pockets ≤3 mm were designated periodontal healthy and were eligible for inclusion in the control group.

### Exclusion criteria

Individuals meeting the following criteria were not eligible for study participation: Less than ten natural teeth, age <18 years, pregnancy or breastfeeding, infectious disease, systematic periodontal therapy within the last five years, antibiotic medication within the last six months, intellectual inability to fully comprehend the aims of the study, severely decayed teeth, atrial fibrillation, or severe cardiac valve vitium.

### Screening, recruitment and examination

A total of 724 subjects were screened, 256 met the inclusion criteria. Ultimately 158 subjects (75 male/83 female) were enrolled in the study. 92 patients were assigned to the test group (severe periodontitis). 74 received the diagnosis of severe chronic periodontitis and 18 suffered from aggressive periodontitis. 66 subjects served as periodontally healthy controls. Individuals meeting the inclusion criteria after the screening examination were informed about the aims of the study and asked for their participation. All patients who participated in the study signed informed consent and received an assessment of their periodontal health by recording a comprehensive periodontal status as well as an assessment of their cardiovascular status by using the Arteriograph within the next seven days. For matching purposes furthermore age, gender, weight, height, smoking habits and the presence of known systemic medical problems (e.g. diabetes) were recorded using a questionnaire.

### Background factors

After setting the age of the evaluated subjects to older than 35 years it was not necessary to match the patients in the sense of pair wise assignment. The limit of 35 years was chosen because in the periodontitis group no one was younger than 36. After this exclusion, except the peripheral pulse pressure, there were no significant differences between the periodontally healthy controls and the test group suffering from severe periodontal diseases regarding the following parameters: gender, age, body mass index, height, weight, smoking habits, arterial hypertension, and presence of hypercholesterolemia. The same holds true for medication (tables 1 and 2).

**Table 1:** Clinical characteristics of the studied population.

		Group		
	Unit	no/mild periodontitis	severe chronic/aggressive periodontitis	P
		n = 66	n = 92	
Age	years	55±13	55±10	0.71
BMI	kg/m^2^	25±4	26±3	0.60
Male gender	%	43,9	50,0	0.45
Smoker	%	30,3	21,7	0.22
Hypertension	%	17.2	22.5	0.44
Hypercholesteremia	%	10.0	10.9	1.0

*Values are expressed as mean ± SD as a result of Mann-Whitney U-Test for age and BMI or in % as a result of Chi-squared for the others.*

**Table 2:** Medication of the studied population.

		Group		
	Unit	no/mild periodontitis	severe chronic/aggressive periodontitis	P
		n = 66	n = 91	
Diuretics (treated)	%	0	3.3	0.29
Statins (treated)	%	8.8	12.0	0.54
β-Blockers (treated)	%	7.0	16.3	0.086
Calcium channel blockers (treated)	%	3.5	3.3	1.0
NSAID (treated)	%	17.5	14.1	0.58
ACE-inhibitors (treated)	%	8.8	15.2	0.24

*Values are expressed in % as result of Chi-squared test. NSAID = non-steroidal anti-inflammatory drugs; ACE = angiotensin converting enzyme.*

### Periodontal examination

The periodontal examination comprised the following parameters: Number of intraoral visible teeth, pocket probing depth (PPD) and clinical attachment level (CAL). Measurements were performed at six sites per tooth using a CP-12 Marquis periodontal probe (Hu Friedy Co., USA); Recorded measurements were rounded to the nearest millimeter. All periodontal examinations were executed by a single clinically experienced dentist. To ensure the reproducibility of the clinical test results, an intra-individual calibration was performed. For this, 4 phantom models with periodontal defects with each 27 teeth were measured at two points in time. The examiner was calibrated if the first measurement and the measurement 48 hours later was were equal at 75% of the measurements with total agreement and 95% within±1 mm [27].

### Vascular examination

After the periodontal examination the recording of the cardiovascular parameters was performed during a subsequent appointment within the next seven days using the Arteriograph (TensioMed Ltd, Hungary) with the corresponding TensioMed analysis software (version 1.9.9.12).

In accordance with the international guidelines for the implementation of arterial stiffness measurements, all measurements were made in the same room under quiet conditions and dim illumination, unaffected by external environmental influences [28].

Firstly the distance between the sternal notch (jugulum) and the symphysis was recorded with a tape measure. Subsequently, in order to minimize sources of recording error each patient had a rest period of ten minutes before the onset of the cardiovascular measurements. All measurements were performed three times, with a predetermined free interval of two minutes between the individual measuring periods. During the examination the study subjects lay relaxed on an examination couch with eyes closed. All vascular data were recorded by the same trained medical technical assistant who was unaware of the assignment of the study subjects to the test or control group.

The analysis of the Arteriograph data was performed by an experienced cardiologist (JB) who also was unaware of the assignment of the data to the different groups.

### Statistical analysis

Statistical data analysis was performed by a professional statistician using the WinMEDAS (C. Grund, Germany) statistical software package.

To calculate the differences between both groups the Mann-Whitney U-test was used for continuous variables. The Chi-square-test or Fishers test for expected values below 5 was used for nominal scale data. The level of significance was set to p<0.05.

## Results

### 1. Pulse Wave Velocity (PWV)

**Figure 2:**
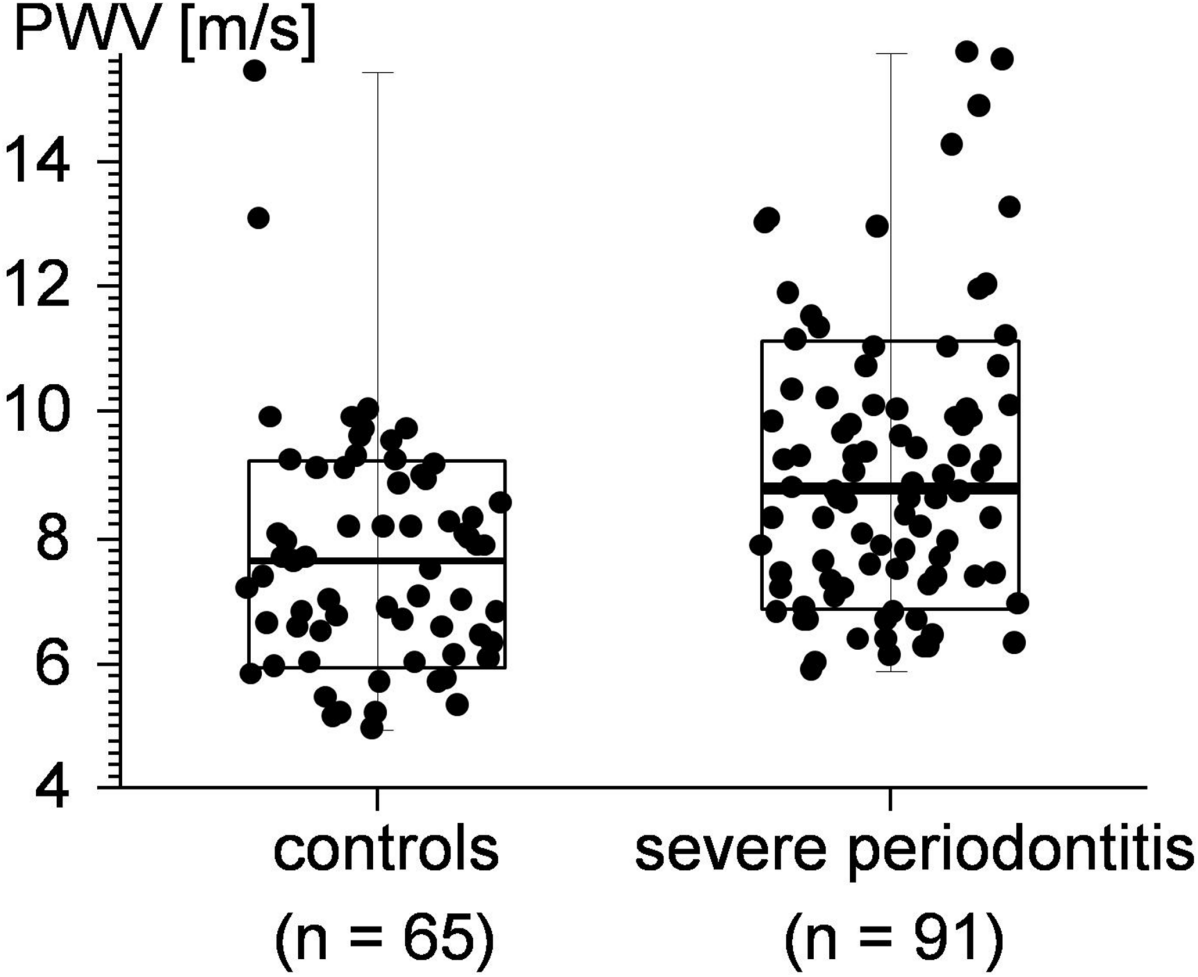
Pulse wave velocity (PWV) data recorded for the 2 experimental groups.

The results of the PWV data analysis are depicted in figure 2. Data evaluation revealed significantly higher PWV values for patients suffering from severe chronic periodontitis or severe aggressive periodontitis when compared to the periodontal healthy controls (p = 0.00004).

### 2. Aortic Augmentation Index (AIx)

**Figure 3:**
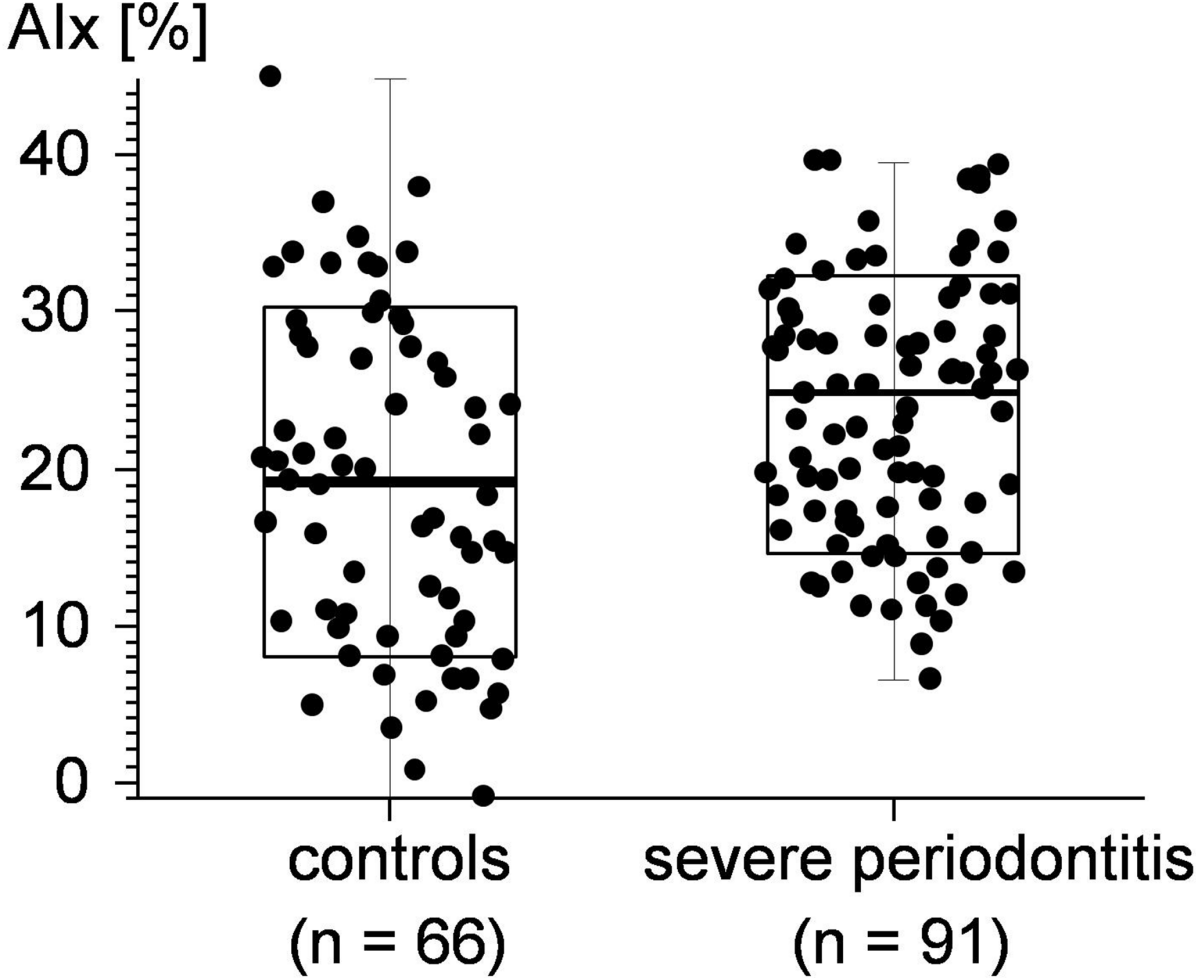
Aortic Augmentation Index (AIx) data calculated for the 2 experimental groups.

The results of the calculation of the AIx data for the test and control group are shown in figure 3. AIx scores calculated for the periodontally healthy individuals of the control group proved to be significantly lower (p = 0.0049) than the AIx scores calculated for those with severe aggressive periodontitis or severe chronic periodontitis.

### 3. Pulse pressure amplification (PPA) and central pressure

In the periodontally healthy controls the peripheral diastolic blood pressure was lower, peripheral systolic blood pressure higher and consequently the peripheral pulse pressure significantly higher (p = 0.0024) than the values recorded for the periodontitis group (see table 3). However when central pulse pressures were compared the differences disappeared, indicating that the pulse pressure and thus the central pressure is higher and pulse pressure amplification lower in the periodontitis group when compared to the healthy controls (p = 0.028 for PPA, table 4). The observed central augmentation pressure shows the tendency to be higher in the periodontitis group (p = 0.064, table 4).

**Table 3:** Hemodynamic characteristics of the studied population.

		Group		
	Unit	no/mild periodontitis	severe chronic/aggressive periodontitis	P
		n = 66	n = 92	
Heart rate	bpm	69±10	67±10	0.089
Peripheral systolic blood pressure	mmHg	134±21	129±16	0.18
Peripheral diastolic blood pressure	mmHg	80±11	82±10	0.89
Peripheral pulse pressure	mmHg	54±14	47±10	0.0024**

*Values are expressed as mean ± SD as a result of Mann-Whitney U-Test.*

**Table 4:** Stiffness- and pulse wave reflection related hemodynamics of the studied population.

		Group		
	Unit	no/mild periodontitis	severe chronic/aggressive periodontitis	P
		n = 66	n = 91	
Central systolic blood pressure	mmHg	125.9±22.9	124.3±17.8	0.87
Central pulse pressure	mmHg	46.2±16.3	42.1±11.3	0.15
Pulse pressure amplification		1.2±0.2	1.1±0.1	0.028*
Aortic augmentation index (AIx)	%	19.1±10.6	23.7±8.3	0.0049**
Augmentation pressure	mmHg	9.9±8.8	10.4±5.8	0.064
Pulse wavevelocity (PWV)	m/s	7.7±1.9	9.1±2.2	0.00004***

*Values are expressed as mean ± SD as a result of Mann-Whitney U-Test.*

## Discussion

The main finding of this study is that in patients suffering from severe chronic or aggressive periodontitis arterial stiffness and pulse wave reflection are significantly increased.

It further supports the evidence for an association between periodontal and cardiovascular health and is in line with the data of several other studies [1], [29]–[32].

The specific relationship between arterial stiffness and periodontitis was documented only once before in a subgroup of patients suffering from arterial hypertension [33]. The data of that study failed to prove a difference in PWV. They revealed a significantly higher left ventricular hypertrophy and significant differences in pulse wave reflection including increased central aortic pressures and increased augmentation in the periodontal disease group in the situation of arterial hypertension. A correlation between the pulse wave velocity and oral inflammation was, by contrast to our data, not confirmed. In terms of pulse wave reflection the results are in concordance with the findings of this study. We were able to identify higher AIx scores in the study subjects suffering from severe periodontal disease when compared to the periodontally healthy controls. Besides a higher sample rate in our study the main difference between Franek’s and our study is patient selection. As in many other studies the study by Franek et al. 2009 evaluated a highly selected population. Only patients with hypertension were included in their trial. Other studies have shown that hypertensive patients themselves display higher pulse wave velocity scores than patients with normal blood pressure [14], [34]. That may be one reason for the diverging study results. Differences induced by hypertension might have superimposed changes caused by periodontal inflammation. Whereas Franek focused on a subgroup of hypertension we included a wide range of cardiovascular compromised as well as cardiovascular healthy subjects. The higher number of evaluated individuals (n = 724) in the present study allowed for a meaningful statistical analysis.

Nevertheless, data analysis revealed that the mean peripheral blood pressure of both groups is within the normal range (≤140 systolic, and ≤90 diastolic). On the basis of a routine peripheral blood pressure control by the method of Riva-Rocci, the average individual in the test group would have been classified as patient with no risk factor for cardiovascular events. This illustrates the potential benefits of the oscillometric pulse wave analysis (e.g. Arteriograph) compared to ordinary blood pressure measurements for the detection of early or preclinical signs of cardiovascular dysfunction.

Another point to discuss is the diagnosis and thus the grouping of the patients. The use of the established Community Periodontal Index of Treatment Needs CPITN [35], for classifying the severity of periodontal disease as done in many previous investigations including the study by Franek et al. should be questioned critically. The scoring system of the CPITN tends to overestimate the severity of periodontal disease expression [1], [36]. For this reason, in all study participants we recorded a comprehensive periodontal status and the severity of disease expression was classified following the proposal of the working group of the Centers for Disease Control (CDC) and the American Academy of Periodontology (AAP) [26]. To ensure the inclusion of individuals with a substantial inflammatory burden in the periodontitis group, only subjects with a minimum of six teeth displaying pockets depth ≥6 mm were recruited, which is also in contrast to the disease severity of patients evaluated in other preceding investigations.

Particularly in young individuals with a diagnosis of aggressive periodontitis the recorded pulse wave velocity values were significantly higher than those found in the periodontally healthy controls (data not shown). This is consistent with findings from other studies documenting a much stronger association between periodontal infections and vascular endpoints in younger patients [37], [38]. By which mechanisms the observed vascular changes are correlated with the manifestation of severe periodontal disease may not be answered by the present data.

“Translating” the arterial stiffness measurements observed in this study into a biological vascular age may serve as an illustration for the importance of the present findings [39]. For this purpose, the results of the present study are compared to standard values obtained from measurements made in the healthy general population. Patients with aggressive periodontitis or severe chronic periodontitis, displaying an average pulse wave velocity of 9.1 m/s their biological vascular age is about 20 years higher than that of the healthy age-corrected group and therefore pre-aged to a huge extent. Even more dramatic are is the interpretation of the results in terms of mortality and the incidence of cardiovascular events. An increase in pulse wave velocity of 1 m/s implies an estimated 14% increased risk for cardiovascular events, and a 15% increased risk for overall mortality rate accordingly to mortality rates of Vlachopoulos’ review [17].

For the prediction of cardiovascular events the central pressure is more important than the peripheral [40]. Again, the present study revealed that periodontitis patients exhibit significantly higher central pressures (expressed as PPA) than periodontal healthy controls reflecting the higher cardiovascular risk of the periodontitis patients. Increased PWV as marker of stiffening of the large arteries suggest that periodontitis patients suffer from a broad range of subclinical vasculature dysfunction.

The most frequently cited reason for the intake of beta-blocker is the diagnosis “hypertension”. Beta-blockers per se (possible exception nebivolol) affect arterial stiffness possibly indirectly by lowering of peripheral blood pressure and/or the initially ejected pressure wave without changing central hemodynamics. Though beta-blockers are known to lower PWV the group with the more frequent intake of β-blockers is showing higher PWV (the periodontitis group). One might speculate that if the intake of beta-blockers would be completely uniformly distributed, the difference in PWV of the groups could even be more pronounced. The same holds true for PPA. Nevertheless both, the intake of beta-blockers and the diagnosis hypertension, are not significantly different in both groups.

This cross-sectional study shows for the first time a pathophysiological and highly significant predictive risk marker of PWV in addition to the above described pulse wave reflection to be higher in patients suffering from severe periodontitis. Further intervention studies using these markers of vascular dysfunction and vascular structure as endpoints may be required to elucidate possible causal relationships between periodontal and cardiovascular disease.
